# Supplementary material for: Signal execution modes emerge in biochemical reaction networks calibrated to experimental data
Source: iScience. 2024 May 16;27(6):109989. doi: 10.1016/j.isci.2024.109989 (PMC11154230; doi:10.1016/j.isci.2024.109989)
Supplement: Document S1. Figures S1–S11 and Tables S1–S5 [file mmc1.pdf]

iScience, Volume *27*

Supplemental information

Signal execution modes emerge in biochemical reaction networks calibrated to experimental data

Oscar O. Ortega, Mustafa Ozen, Blake A. Wilson, James C. Pino, Michael W. Irvin, Geena V. Ildefonso, Shawn P. Garbett, and Carlos F. Lopez

Supplemental Information

1 Reducing execution mode uncertainty through parameter measurements

Given that the aEARM calibrated parameter vectors yield three execution modes (Figure 2C-D in the main text) with their respective probabilities, there is uncertainty about which execution mode is most representative of the cellular process. We then asked whether we could identify parameters that, if measured experimentally, would reduce the execution mode uncertainty. We hypothesize that identifying key parameters that inform execution mechanisms could guide experiments to improve our knowledge about network-driven signal processing. To measure the uncertainty of the execution modes, we used Shannon's entropy[1] formulated as $H = -\sum_{i=1}^{n} P(x_i)\log_2 P(x_i)$. As aEARM has three execution modes, the maximum entropy in the system is $\log_2 3 = 1.58$, which would signify that each execution mode has a 33% probability. Using the probabilities of the previously obtained execution modes (Figure 2C in the main text) and Shannon's formula, we calculated an entropy of 1.54 indicating a high uncertainty in the execution across all modes. To determine the most informative parameters that should be measured to reduce execution mode uncertainty, we used XGBOOST[2], a gradient-boosted Machine Learning technique that can classify parameter vectors into their corresponding execution modes. We used the calibrated parameter sets as training data where each kinetic parameter (kf, kr, kc, i.e., forward, reverse, and catalysis reaction constants, respectively) is a feature, and the mode of execution is our target variable.

Feature importance analysis from the XGBOOST analysis shows that the model parameters kf6 and kf7 contribute the most to training loss reduction during the classification task (Figure S6A). As illustrated in Figure S6C, parameters kf6 and kf7 correspond to the binding rate of MOMP* to inactivated MOMP, and MOMP* to eC, respectively. These two parameters are part of the reactions where the signal flux is bifurcated in the network, indicating that their values play an important role in the definition of the execution modes. To show the differences in parameter values for each execution mode we plotted the values of the kf6 and kf7 parameters. As shown in Figure S6B the execution modes have different distributions of the kf6 and kf7 parameters with some overlap. To show how measuring the kinetic parameters, the binding rate of MOMP* to eC, reduces the uncertainty in the mode of signal execution, we simulated 100 measurements of the kf7 parameter, and calculated the entropy with the updated execution modes probabilities. As shown in Figure S6D, measurements in the range of -8.1 and -7.15, where there is no overlap between the parameter distributions, reduce the entropy to less than 0.05 which indicates that we know the execution mode with certainty. For measurements larger than -7.15 but lower than -5.37 the overlap between distributions starts to increase and with it the uncertainty increases as well, as indicated by the entropy. Then, for measurements larger than -5.37 the entropy starts to decrease. This indicates that in general measuring kinetic

parameters reduces the entropy and thus the uncertainty in the execution mode with the exception where the overlap between distribution is maximum and increases the entropy.

2 Modes of signal execution in a detailed apoptosis model with increased parameter uncertainty

Based on our results with aEARM, we then asked how a larger model with higher parameter uncertainty would fare under the presented signal execution analysis. We shifted to a larger extrinsic apoptosis reaction model (EARMv2.0), which has been studied and characterized in previous work[3]. As illustrated in Figure S7A, EARMv2.0 is considerably larger than aEARM as the biochemical interactions are described with higher molecular resolution. In all, EARMv2.0 has 77 molecular species and 105 kinetic parameters. As described in Methods, we used PyDREAM to calibrate the model to published experimental data[4]. Although the calibration yielded parameter vectors that fit the experimental data indistinguishably well (Figure S8), we note that only 62 model parameters converged according to the Gelman-Rubin diagnostic (GR < 1.2) after two million iterations (see Table S5 and Figure S9). Distributions of 9 converged parameters are shown in Figure S7B. The remaining parameters exhibited GR values between 1.21 and 13.52 (Table S4 and Figure S10a, b, and c). From a Bayesian perspective, non-convergent parameters imply that the experimental data simply cannot constrain their values to a distribution and thus results in higher variability. As our analysis is focused on understanding execution modes in network-driven processes, a model with poorly identified parameters presents an opportunity to explore how signal execution could be best interpreted and understood in large model systems with high parameter uncertainty.

We followed the same procedure used in the main text to explore the execution modes in EARMv2.0 (See Methods for details). Our analysis shows that calibrating EARMv2.0 to experimental data constrains the signal flow to eleven execution modes that can be represented by 53 dominant subnetworks out of 2067 possible subnetworks. As shown in Figure S7C, the apoptosis execution signal could flow through any of these paths with varying degrees of probability, with Execution Mode 1 (EM1) exhibiting a probability of ~20% and the first four modes capturing ~50% of the signal probability, thus suggesting high path entropy as we have seen in previous work[5]. Supplemental videos show animations of signal flow for all execution modes in the context of EARMv2.0.

If one needs to compare EMs observed for aEARM and EARMv2.0, we see that there exists an overlap between the underlying highly probable dominant pathways although they visually seem different. The main difference in the EMs is that complex interactions in EARMv2.0 are coarse-grained into a single node in aEARM and thus the EMs appear simplified. For instance, the MOMP node in aEARM represents the mitochondrial outer membrane pore formation which is explicitly implemented in EARMv2.0 by the Bid to Smac pathways. Thus, the EMs obtained from aEARM and the EMs obtained from EARMv2.0 are comparable and comprise functional commonality. While the EARM models represent the same mechanism, their

resolution and, hence, the underlying system of differential equations are different. Therefore, the functional commonality in the observed EMs may imply that the EMs are intrinsic features of the underlying system and independent of the specific way in which the model is implemented. Additionally, having more uncertain parameters, as well as a larger network size, results in more execution modes in EARMv2.0. Therefore, we believe the number of EMs would be related to the network size rather than the amount of data because as the size of the network increases, the number of possible pathways playing a role in the production (or consumption) of the target node might increase as well, which may result in the identification of more EMs.

Next, we tested whether each execution mode exhibits different responses to the same perturbation. We selected EM1 and EM2 for analysis as these modes exhibit the highest probability for signal execution. As illustrated in Figure S7D, the mBid interaction with Mcl1 is dominant in EM1. In contrast, the mBid interactions with both Mcl1 and Bcl2 are dominant in EM2, thus highlighting the importance of both antiapoptotic proteins to understand the signaling mechanisms during apoptosis execution when the cell is exposed to an apoptotic inducer.

We then performed two *in silico experiments* for EM1 and EM2: (i) a 50% knockdown (KD) of the antiapoptotic protein Mcl1 as well as (ii) a 50% knockdown of the antiapoptotic protein Bcl2. For the Mcl1 KD, we found that the EM1 median Time of Death (ToD) decreased from 10022.46 s (WT) to 8686.52 s (Figure S11A-upper panel). This is expected since mBid and Mcl1 interactions are dominant in this mode. By contrast the median ToD in EM2 decreased from 9943.65 s (WT) to 9335.85 s. This modest decrease in ToD can be attributed to the fact that although Mcl1 and mBid interactions are important in EM2, the dominance of Bcl2 compensates for the absence of Mcl1 and reduces the impact on ToD for the Mcl1 KD.

For the Bcl2 KD, we found that the median ToD in EM1 has a minor change from 10022.46 s to 10011.87 s, as expected because mBid activity is not significantly affected by Bcl2 in this mode. By contrast, in EM2, mBid activity is modulated by Mcl1 and Bcl2. Thus, a reduction in the initial protein levels of Bcl2 enables more mBid proteins to activate pro-apoptotic proteins and this leads to an increase in ToD to 9580 s (Figure S11A-upper panel). Taken together this data shows that distinct execution modes respond differently to the same perturbation and that their responses can be predicted based on the dominant reactions for a given execution mode.

To further emphasize the importance of transient dynamics on signal processing, we explored EM1 dynamic fingerprints and found that SMAC inhibition of XIAP occurs at later time points of the simulations (>8640 s). Therefore, we hypothesized that XIAP inhibition would be more effective earlier during signal execution. To test this, we added an XIAP inhibitor to EARMV2.0 at either 4000 s or 8000 s. As shown, when the inhibitor is added at the later time point, we observed a small reduction (Figure S11A lower panel) in the median ToD from 9943.65 s in the WT to 9380.44 s (Δt = 563.21 s). In contrast, when the inhibitor is added at the earlier time

point, when SMAC is not yet released from the mitochondria, the inhibitor binds to XIAP enabling C3 to cleave PARP and thereby reducing the median ToD to 6766.10 (Δt = 3177.55 s).

As combination therapies have become important to combat drug resistance[6,7] , especially in cancers, we explored whether our analysis provided information about potential targets for co-treatment. As we previously mentioned, Mcl1 and XIAP are dominant antiapoptotic proteins in EM1, thus we hypothesized that inhibition of both proteins would yield a shorter ToD compared to only inhibiting XIAP. To test this, we added two drugs that independently inhibit XIAP and Mcl1 and obtained a ToD of 5951.11 s representing a 12% reduction in the ToD compared to XIAP inhibition only (Figure S11A lower panel). Finally, to guide experiments that would identify the most likely execution mode out of the 11 execution modes obtained, we developed an XGBOOST model of execution mode estimation and performed feature importance analysis. As shown in Figure S11B, we found that the parameters controlling the kinetics of mBid binding to BcxL, and XIAP binding to C3 yield the most information about execution modes in EARMv2.0. Taken together, these results suggest that the analysis of signaling dynamics from uncertain parameters helps us identify dominant reactions that control signal flow in a network during signal processing and how these networks are more sensitive to the perturbations of those reactions.

3 Tables

Table S1. Reactions in aEARM, related to Figure 1A. *"*" indicates the activated state of the species. ":" indicates complexation. "⇌" indicates reversible reactions, whereas "⟶" indicates forward reactions.*

**Reaction**	**Parameters**
TRAIL + R ⇌ TRAIL:R ⟶ DISC	kf0, kr0, kc0
DISC + iCaspases ⇌ DISC:iCaspases ⟶ DISC + *iCaspases	kf1, kr1, kc1
*iCaspases + eCaspases ⇌ *iCaspases:eCaspases ⟶ *iCaspases + *eCaspases	kf2, kr2, kc2
*eCaspases + iCaspases ⇌ *eCaspases:iCaspases ⟶ *eCaspases + *iCaspases	kf3, kr3, kc3
*iCaspases + Bid ⇌ *iCaspases:Bid ⟶ *iCaspases + tBid	kf4, kr4, kc4
tBid + MOMP_Signals ⇌ tBid:MOMP_Signals ⟶ tBid + *MOMP_Signals	kf5, kr5, kc5
*MOMP_Signals + MOMP_Signals ⇌ *MOMP_Signals:MOMP_Signals ⟶ *MOMP_Signals + *MOMP_Signals	kf6, kr6, kc6
*MOMP_Signals + eCaspases ⇌ *MOMP_Signals:eCaspases ⟶ *MOMP_Signals + *eCaspases	kf7, kr7, kc7
*eCaspases + PARP ⇌ *eCaspases:PARP ⟶ *eCaspases + cPARP	kf8, kr8, kc8
iCaspases ⟶ ∅	kc9

Table S2. ODEs of aEARM, related to Figure 1A. *Only the equations for the individual species are provided, and the equations for the intermediate species are omitted.*

**Reaction**
$\frac{d[\mathrm{TRAIL}]}{dt} = kr0[\mathrm{TRAIL:R}] - kf0[\mathrm{TRAIL}][\mathrm{R}]$
$\frac{d[R]}{dt} = kr0[\mathrm{TRAIL:R}] - kf0[\mathrm{TRAIL}][\mathrm{R}]$
$\frac{d[\mathrm{DISC}]}{dt} = kc0[\mathrm{TRAIL:R}] + kr1[\mathrm{DISC:iCaspases}] + kc1[\mathrm{DISC:iCaspases}] - kf1[\mathrm{DISC}][\mathrm{iCaspases}]$
$\frac{d[\mathrm{iCaspases}]}{dt} = kr1[\mathrm{DISC:iCaspases}] + kr3[*\,\mathrm{eCaspases:iCaspases}] - kf1[\mathrm{DISC}][\mathrm{iCaspases}] - kf3[*\,\mathrm{eCaspases}][\mathrm{iCaspases}]$
$\frac{d[\mathrm{eCaspases}]}{dt} = kr2[\mathrm{eCaspases:*\,iCaspases}] + kr7[\mathrm{eCaspases:*\,MOMP_{Signals}}] - kf2[*\,\mathrm{iCapsases}][\mathrm{eCaspases}] - kf7[\mathrm{eCaspases}][*\,\mathrm{MOMP_{Signals}}]$
$\frac{d[\mathrm{Bid}]}{dt} = kr4[\mathrm{Bid:*\,iCaspases}] - kf4[\mathrm{Bid}][*\,\mathrm{iCaspases}]$
$\frac{d[\mathrm{MOMP_{Signals}}]}{dt} = kr5[\mathrm{tBid:MOMP_{Signals}}] + kr6[*\,\mathrm{MOMP_{Signals}:MOMP_{Signals}}] - kf5[\mathrm{tBid}][\mathrm{MOMP_{Signals}}] - kf6[*\,\mathrm{MOMP_{Signals}}][\mathrm{MOMP_{Signals}}]$
$\frac{d[\mathrm{PARP}]}{dt} = kr8[*\,\mathrm{eCaspases:PARP}] - kf8[*\,\mathrm{eCaspases}][\mathrm{PARP}]$

Table S3. Gelman-Rubin (GR) diagnostic values for all calibrated parameters in the aEARM model, related to Figure 1C. *Parameters with GR values lower than 1.2 are considered to be converged.*

Parameter	GR
kf0	1.08514627
kr0	1.00220123
kc0	1.03226655
kf1	1.0526068
kr1	1.00504595
kc1	1.02257591
kf2	1.0130557
kr2	1.00305479
kc2	1.00393523
kf3	1.00236732
kr3	1.00518277
kc3	1.00208664
kf4	1.03636438
kr4	1.00973982
kc4	1.07074804
kf5	1.06039857
kr5	1.11159688
kc5	1.06253039
kf6	1.16079145
kr6	1.01920037
kc6	1.12806401
kf7	1.1978977
kr7	1.015523
kc7	1.04973795
kf8	1.09112645
kr8	1.00130727
kc8	1.05073858
kc9	1.00629721

Table S4. Silhouette score for different numbers of clusters for aEARM, related to Figure 2C.

Number of clusters	Silhouette Score
2	0.696260
**3**	**0.935141**
4	0.886408
5	0.872150
6	0.862335
7	0.828153
8	0.827130
9	0.814062
10	0.810126

Table S5. Gelman-Rubin (GR) diagnostic values for all calibrated parameters in the EARMv2.0 model, related to Supplemental Section 2. *Parameters with GR values lower than 1.2 are considered to be converged*

Parameter	GR
bind_L_R_to_LR_kf	1.30555024
bind_L_R_to_LR_kr	1.19366713
convert_LR_to_DISC_kc	1.59500279
bind_DISC_C8pro_to_DISCC8pro_kf	1.62566521
bind_DISC_C8pro_to_DISCC8pro_kr	1.01651954
catalyze_DISCC8pro_to_DISC_C8A_kc	1.00638202
bind_C8A_BidU_to_C8ABidU_kf	1.57868642
bind_C8A_BidU_to_C8ABidU_kr	1.03076058
catalyze_C8ABidU_to_C8A_BidT_kc	6.63072247
bind_DISC_flip_kf	1.02016009
bind_DISC_flip_kr	1.02119955
bind_BAR_C8A_kf	2.14919922
bind_BAR_C8A_kr	1.00174068
equilibrate_SmacC_to_SmacA_kf	1.01189403
equilibrate_SmacC_to_SmacA_kr	1.03203665
equilibrate_CytoCC_to_CytoCA_kf	1.1986676
equilibrate_CytoCC_to_CytoCA_kr	1.12600228
bind_CytoCA_ApafI_to_CytoCAApafI_kf	1.22739716
bind_CytoCA_ApafI_to_CytoCAApafI_kr	1.02685524
catalyze_CytoCAApafI_to_CytoCA_ApafA_kc	1.03909011
convert_ApafA_C9_to_Apop_kf	2.04271115
convert_ApafA_C9_to_Apop_kr	1.45004061
bind_Apop_C3pro_to_ApopC3pro_kf	3.88094106
bind_Apop_C3pro_to_ApopC3pro_kr	1.46376778
catalyze_ApopC3pro_to_Apop_C3A_kc	2.12805512
bind_Apop_XIAP_kf	1.63051009
bind_Apop_XIAP_kr	1.26680774
bind_SmacA_XIAP_kf	1.05473289
bind_SmacA_XIAP_kr	1.0034777
bind_C8A_C3pro_to_C8AC3pro_kf	3.43426491
bind_C8A_C3pro_to_C8AC3pro_kr	1.01372768
catalyze_C8AC3pro_to_C8A_C3A_kc	1.87635914
bind_XIAP_C3A_to_XIAPC3A_kf	1.67972634

bind_XIAP_C3A_to_XIAPC3A_kr	1.15656166
catalyze_XIAPC3A_to_XIAP_C3ub_kc	1.09385208
bind_C3A_PARPU_to_C3APARPU_kf	1.25018223
bind_C3A_PARPU_to_C3APARPU_kr	1.04884998
catalyze_C3APARPU_to_C3A_PARPC_kc	1.12017976
bind_C3A_C6pro_to_C3AC6pro_kf	13.5217835
bind_C3A_C6pro_to_C3AC6pro_kr	1.03639819
catalyze_C3AC6pro_to_C3A_C6A_kc	1.03041378
bind_C6A_C8pro_to_C6AC8pro_kf	4.69722606
bind_C6A_C8pro_to_C6AC8pro_kr	1.07790728
catalyze_C6AC8pro_to_C6A_C8A_kc	1.120102
equilibrate_BidT_to_BidM_kf	2.61600196
equilibrate_BidT_to_BidM_kr	3.09256962
bind_BidM_BaxC_to_BidMBaxC_kf	3.70912317
bind_BidM_BaxC_to_BidMBaxC_kr	1.02294992
catalyze_BidMBaxC_to_BidM_BaxM_kc	1.07300834
bind_BidM_BaxM_to_BidMBaxM_kf	1.23579073
bind_BidM_BaxM_to_BidMBaxM_kr	1.02038414
catalyze_BidMBaxM_to_BidM_BaxA_kc	1.08771274
bind_BidM_BakM_to_BidMBakM_kf	1.05678249
bind_BidM_BakM_to_BidMBakM_kr	1.18477726
catalyze_BidMBakM_to_BidM_BakA_kc	1.29135272
bind_BaxA_BaxM_to_BaxABaxM_kf	1.44060819
bind_BaxA_BaxM_to_BaxABaxM_kr	1.00404728
catalyze_BaxABaxM_to_BaxA_BaxA_kc	1.02261922
bind_BakA_BakM_to_BakABakM_kf	1.79854806
bind_BakA_BakM_to_BakABakM_kr	1.24688942
catalyze_BakABakM_to_BakA_BakA_kc	1.04152718
bind_BidM_Bcl2M_kf	1.32171588
bind_BidM_Bcl2M_kr	1.25176661
bind_BidM_BclxLM_kf	1.42163672
bind_BidM_BclxLM_kr	1.25451231
bind_BidM_Mcl1M_kf	2.52365114
bind_BidM_Mcl1M_kr	1.53131431
bind_BaxA_Bcl2_kf	1.03114713
bind_BaxA_Bcl2_kr	1.01410852
bind_BaxA_BclxLM_kf	1.48716584
bind_BaxA_BclxLM_kr	1.8534663

bind_BakA_BclxLM_kf	1.09290472
bind_BakA_BclxLM_kr	1.28694699
bind_BakA_Mcl1M_kf	1.03932055
bind_BakA_Mcl1M_kr	1.01668657
bind_BadM_Bcl2_kf	1.00495924
bind_BadM_Bcl2_kr	1.01024336
bind_BadM_BclxLM_kf	1.01820425
bind_BadM_BclxLM_kr	1.01971635
bind_NoxaM_Mcl1M_kf	1.01150003
bind_NoxaM_Mcl1M_kr	1.02564465
assemble_pore_sequential_Bax_2_kf	1.99372138
assemble_pore_sequential_Bax_2_kr	1.0432823
assemble_pore_sequential_Bax_3_kf	1.0519527
assemble_pore_sequential_Bax_3_kr	1.00962278
assemble_pore_sequential_Bax_4_kf	1.013291
assemble_pore_sequential_Bax_4_kr	1.00694199
assemble_pore_sequential_Bak_2_kf	1.24674091
assemble_pore_sequential_Bak_2_kr	1.05713598
assemble_pore_sequential_Bak_3_kf	1.01164877
assemble_pore_sequential_Bak_3_kr	1.03205593
assemble_pore_sequential_Bak_4_kf	1.0717765
assemble_pore_sequential_Bak_4_kr	1.00338011
pore_transport_complex_BaxA_4_CytoCM_kf	1.36126051
pore_transport_complex_BaxA_4_CytoCM_kr	1.0860153
pore_transport_dissociate_BaxA_4_CytoCC_kc	1.19093017
pore_transport_complex_BaxA_4_SmacM_kf	1.21029872
pore_transport_complex_BaxA_4_SmacM_kr	1.02142634
pore_transport_dissociate_BaxA_4_SmacC_kc	1.08568557
pore_transport_complex_BakA_4_CytoCM_kf	1.1615882
pore_transport_complex_BakA_4_CytoCM_kr	1.04931225
pore_transport_dissociate_BakA_4_CytoCC_kc	1.22633259
pore_transport_complex_BakA_4_SmacM_kf	1.33965964
pore_transport_complex_BakA_4_SmacM_kr	1.02048459
pore_transport_dissociate_BakA_4_SmacC_kc	1.01618226

5 Figures

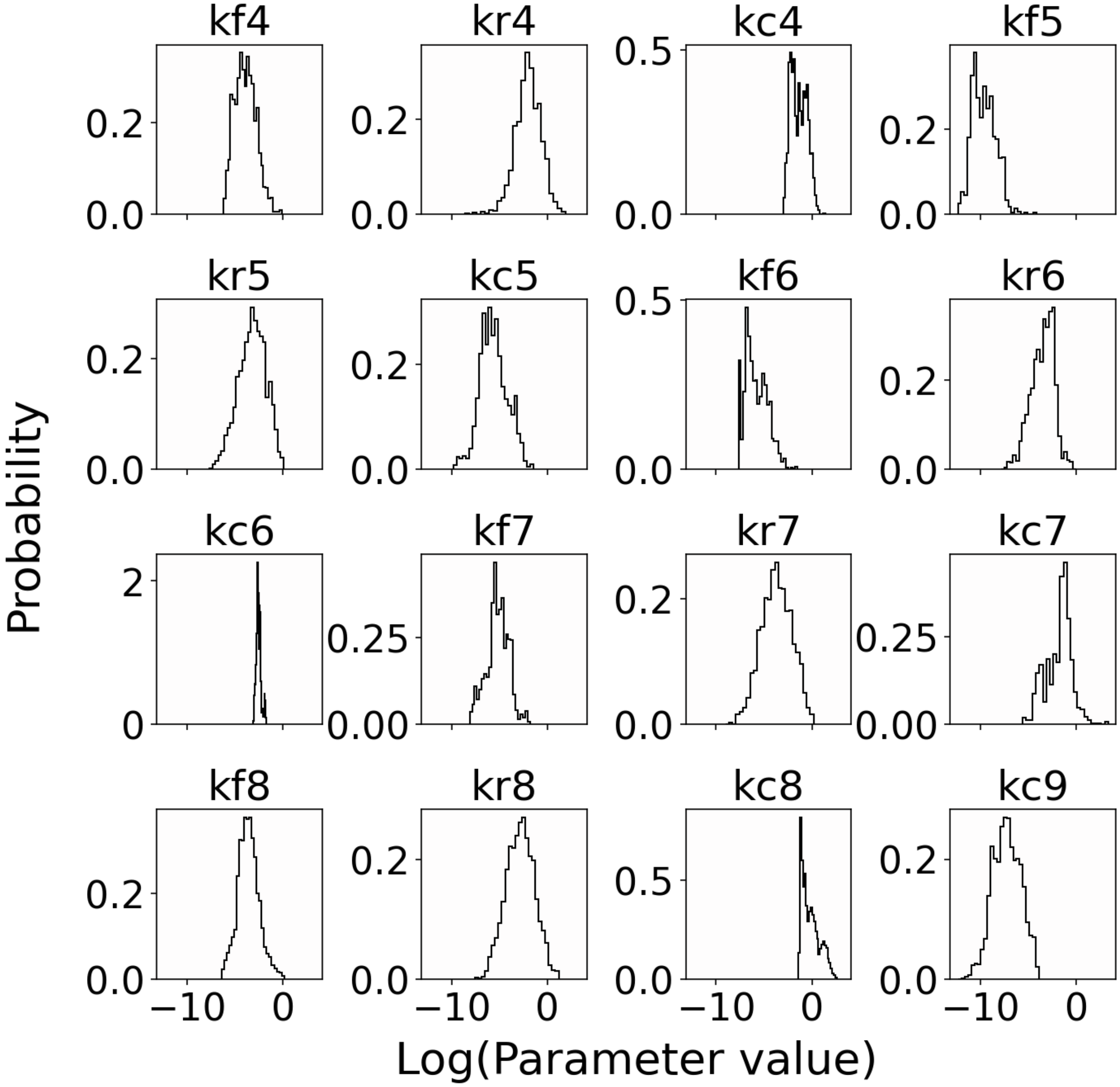

Figure S1. Marginal probability distributions of aEARM individual kinetic parameters, related to Figure 1B. *Distributions were recovered from the PyDREAM run by integrating all other dimensions.*

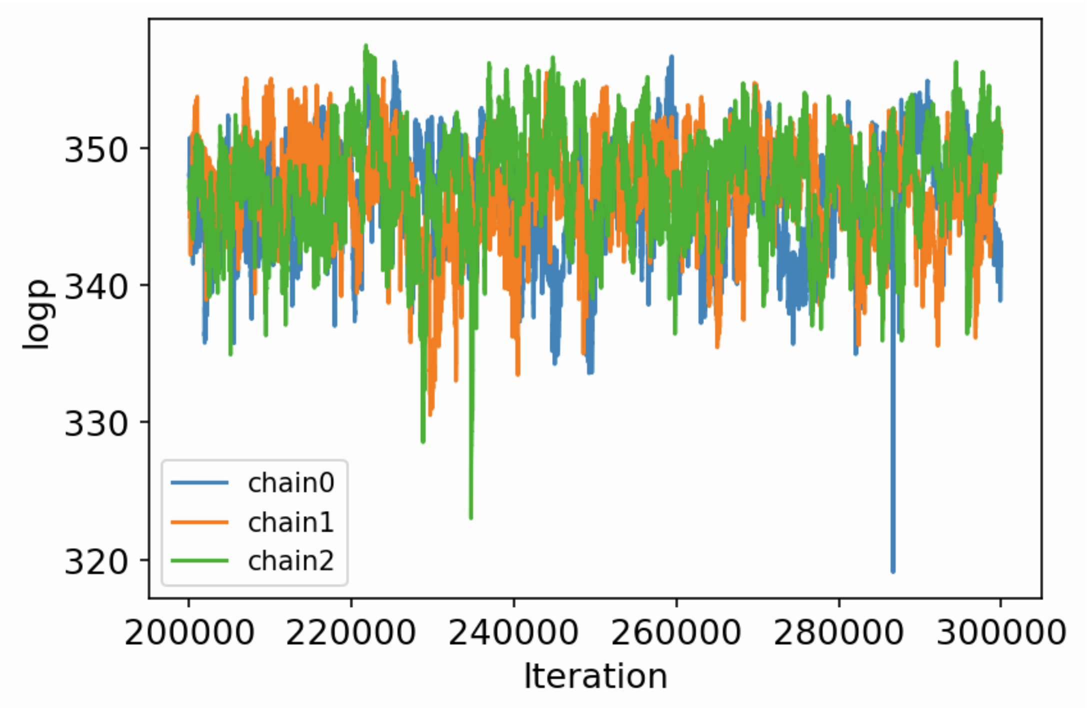

Figure S2. Negative log-posterior values of the posterior distribution of aEARM sampled by 3 chains using PyDREAM after a burn-in of 200,000 iterations, related to Figure 1C.

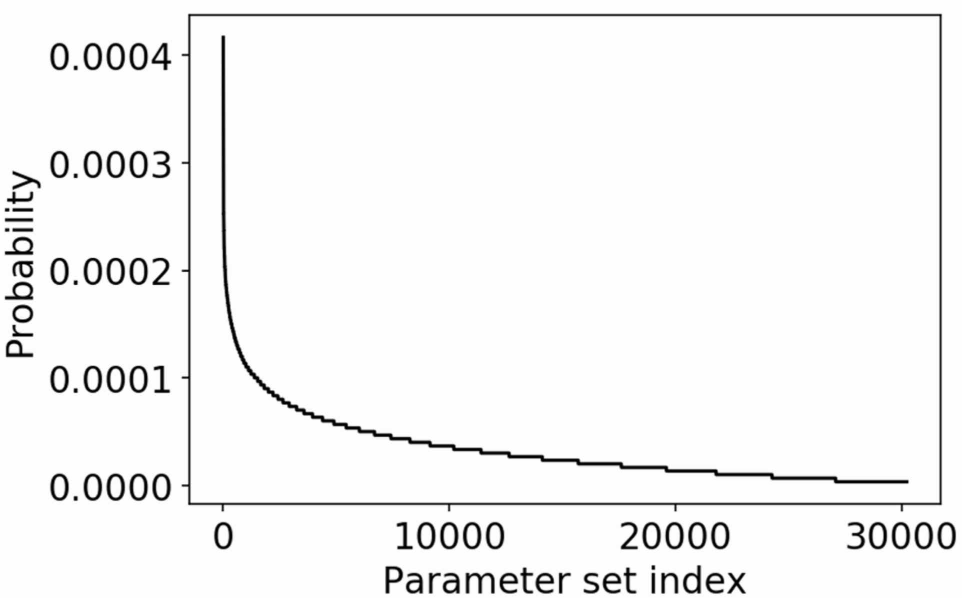

Figure S3. Probability of each of the unique parameter vectors sampled after burn-in in the PyDREAM calibration, related to Figure 1C. *To obtain the probability of each parameter set, the number of visits to a specific parameter vector was normalized by the total number of visits.*

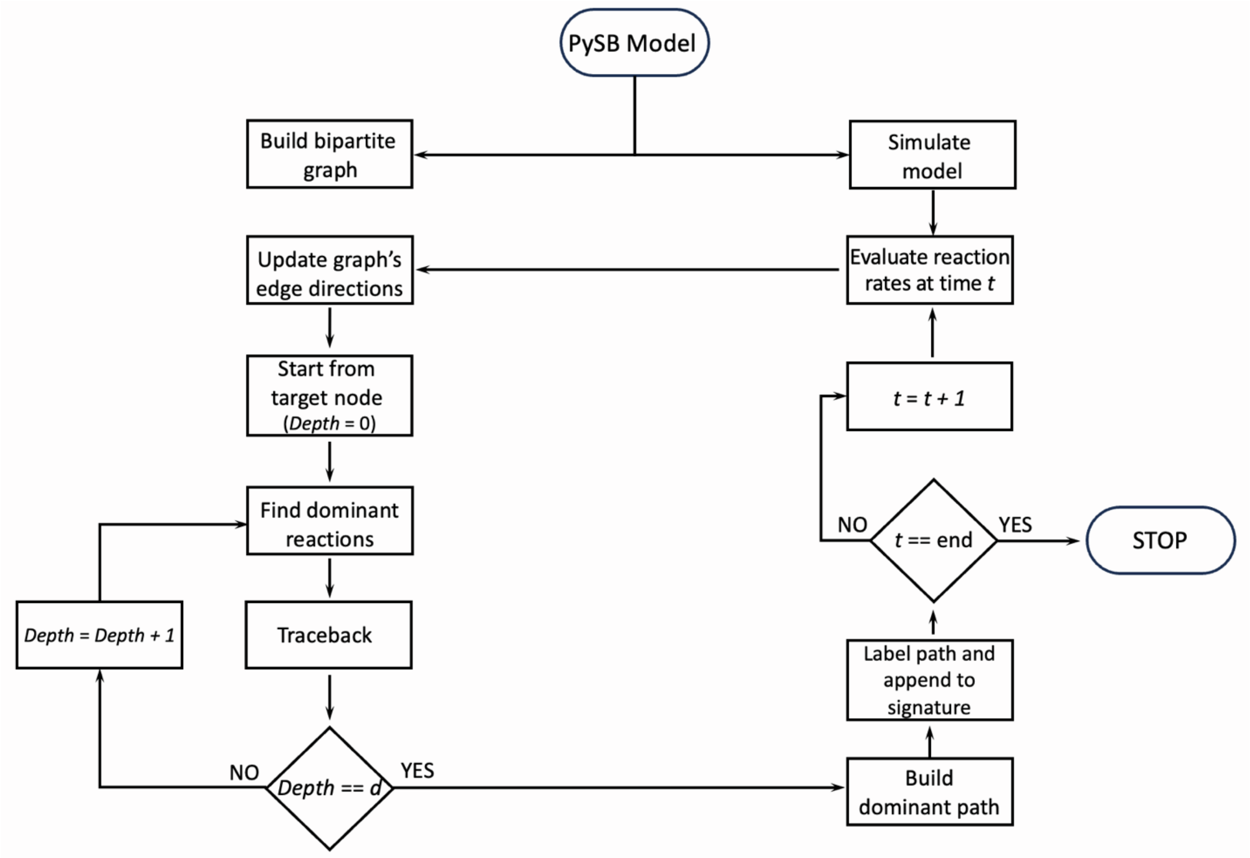

Figure S4. Algorithm to obtain dynamic fingerprints from a simulation, related to Figure 2 and STAR Methods. *Briefly, the algorithm consists in building a bipartite graph from the model, and then simulating the model with a specific parameter vector. Next, the algorithm obtains the dominant reactions from a network and builds a subnetwork. This procedure is repeated for each simulated time point.*

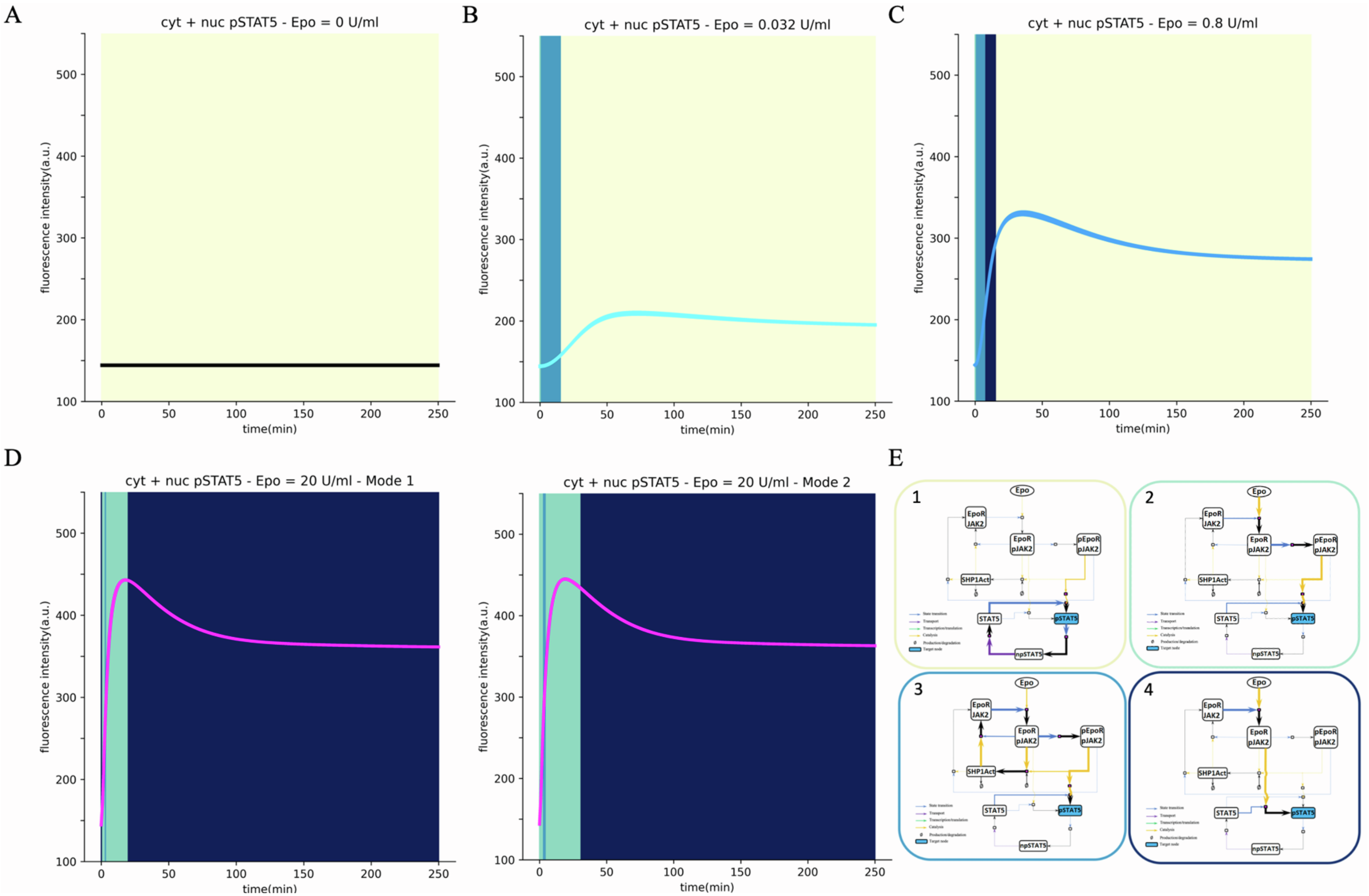

Figure S5. Dominant pathways over time versus the pSTAT5 abundance with respect to increasing Epo doses, related to Figure 5. *A, B, C, and D) Dominant pathways versus pSTAT5 trajectories when Epo = 0 U/ml, 0.032 U/ml, 0.8 U/ml, and 20 U/ml, respectively. E) Dominant pathways over time framed with the corresponding colors in A-D.*

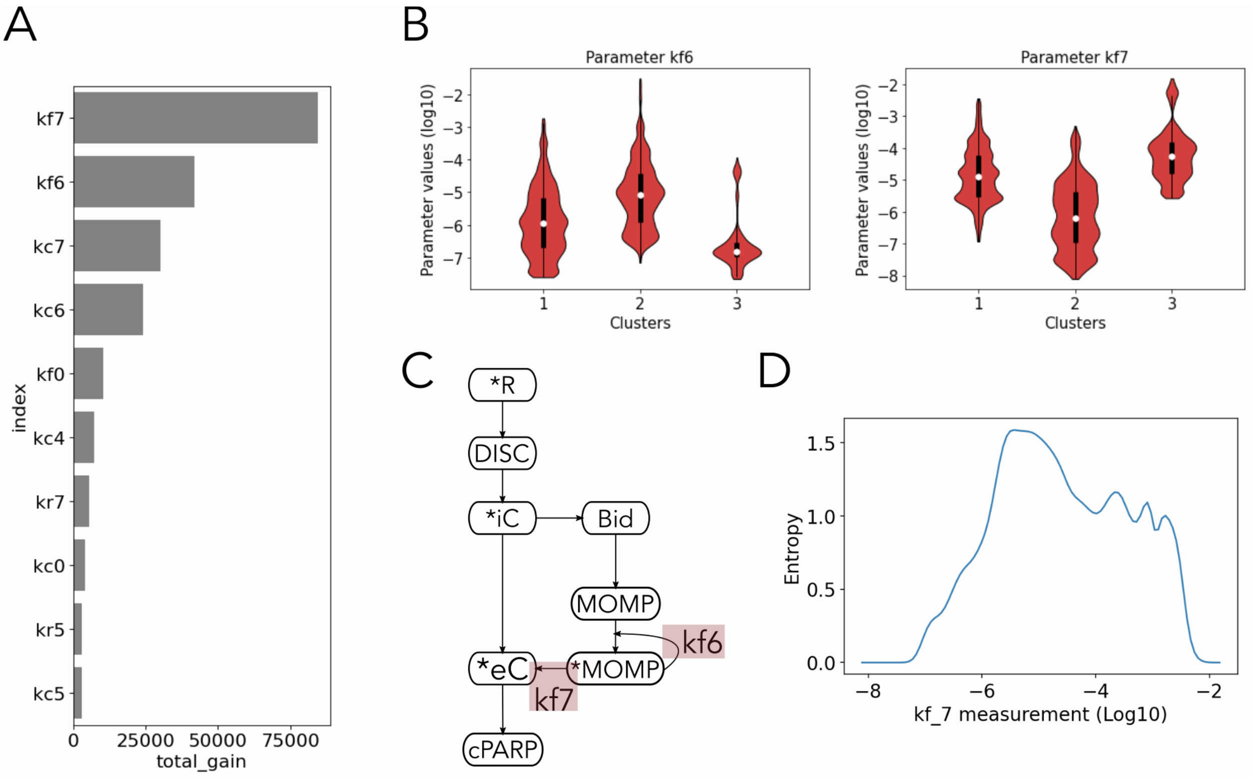

Figure S6. Parameter measurements reduce aEARM execution mode uncertainty, related to Supplemental Section 1. *A) List of the 10 parameters that contribute the most to model prediction. Parameters with higher total gains, compared to another parameter, provide larger improvements to accuracy in model prediction. B) Parameter values of kf6 and kf7 grouped by the execution mode they belong to. A Gaussian kernel was used to estimate the density probability of parameter values in each execution mode. C) Schematic representation of the aEARM network. Kinetic parameters kf6 and kf7 and their corresponding reactions are highlighted in the network. D) Changes in the execution modes entropy after simulated measurements of kf7.*

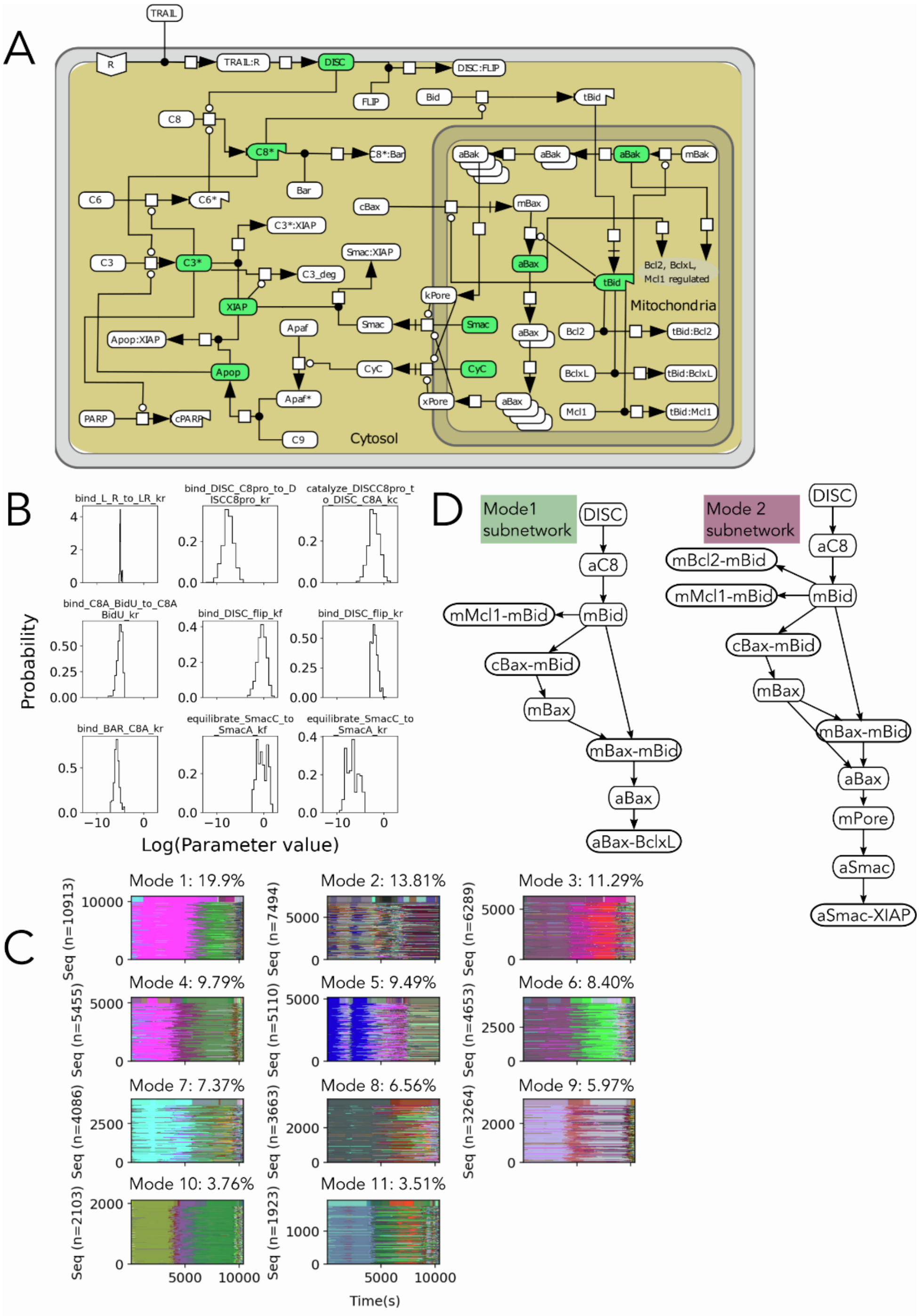

Figure S7. Modes of signal execution in the full Extrinsic Apoptosis Reaction Network, related to Supplemental Section 2. *A) Network of the interactions between the proteins in the apoptosis pathway. Proteins highlighted in green are nodes where the signal flux can be divided. The convention of Kitano*[8] *was followed. B) Marginal probability distributions of 9 individual kinetic parameters converged by the Gelman-Rubin diagnostic. C) Dynamic fingerprints organized by the execution modes they belong to. Each cluster plot is composed of horizontal lines that correspond to dynamic fingerprints, i.e. sequences of dominant subnetworks, and each subnetwork is assigned a different color. Execution modes are sorted from highest to lowest probability. D) Signal execution in Mode 1 (left) and Mode 2 (right) as defined by the most common subnetwork in each mode at t=7000s.*

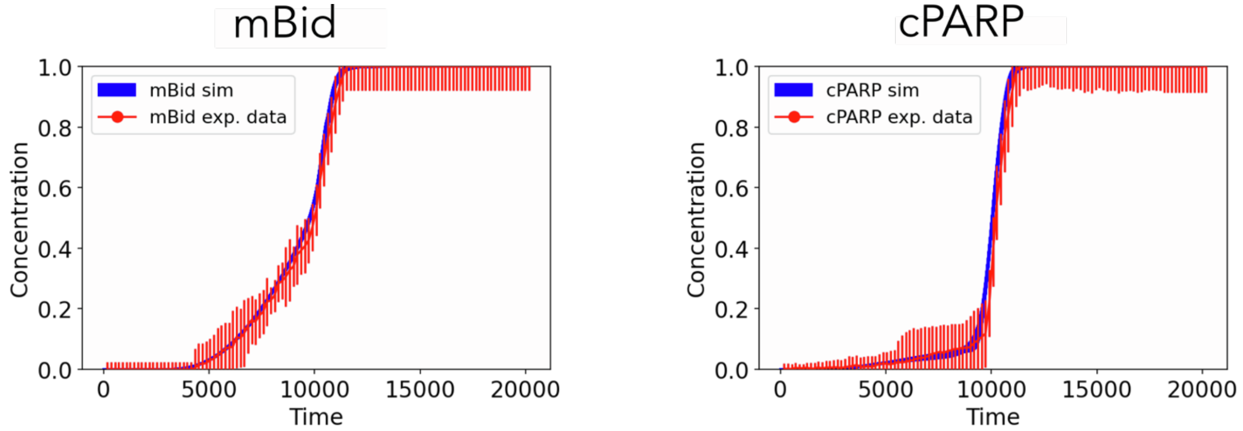

Figure S8. Fitness of EARMv2.0 to experimental data, related to Supplemental Section 2. *Simulated trajectories of truncated Bid and Cleaved PARP calibrated to reproduce the experimental data. Red dots and bars indicate the mean and standard deviation of the experimental data and blue lines correspond to the simulated trajectories.*

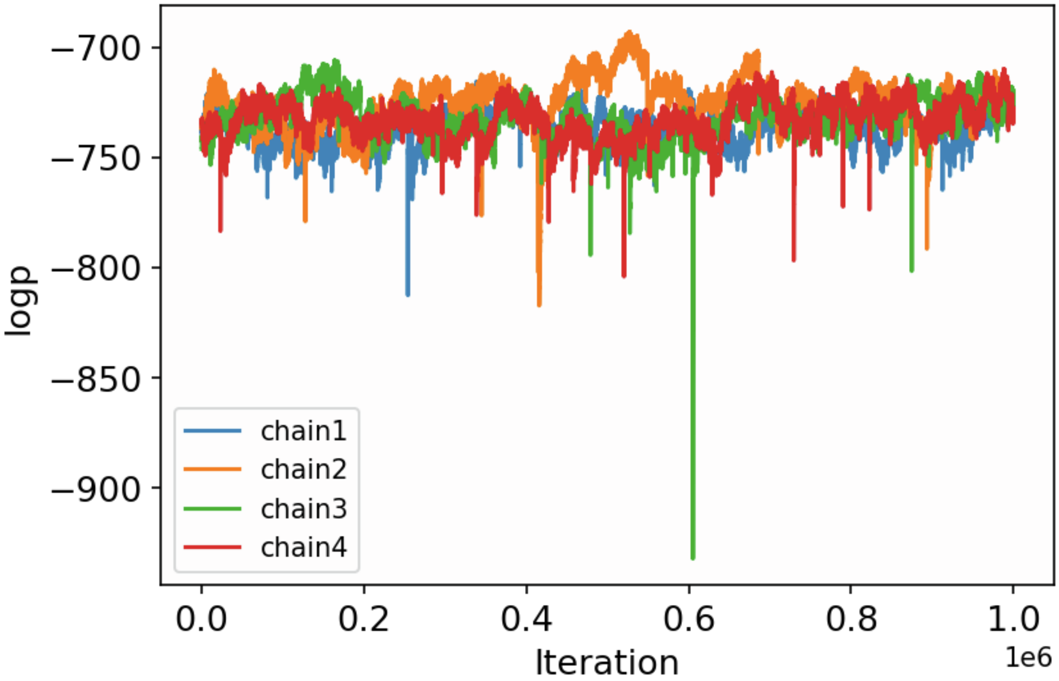

Figure S9. Negative log-posterior values of the posterior distribution of EARMv2.0 sampled by 4 chains using PyDREAM after a burn-in of 1 million iterations, related to Supplemental Section 2.

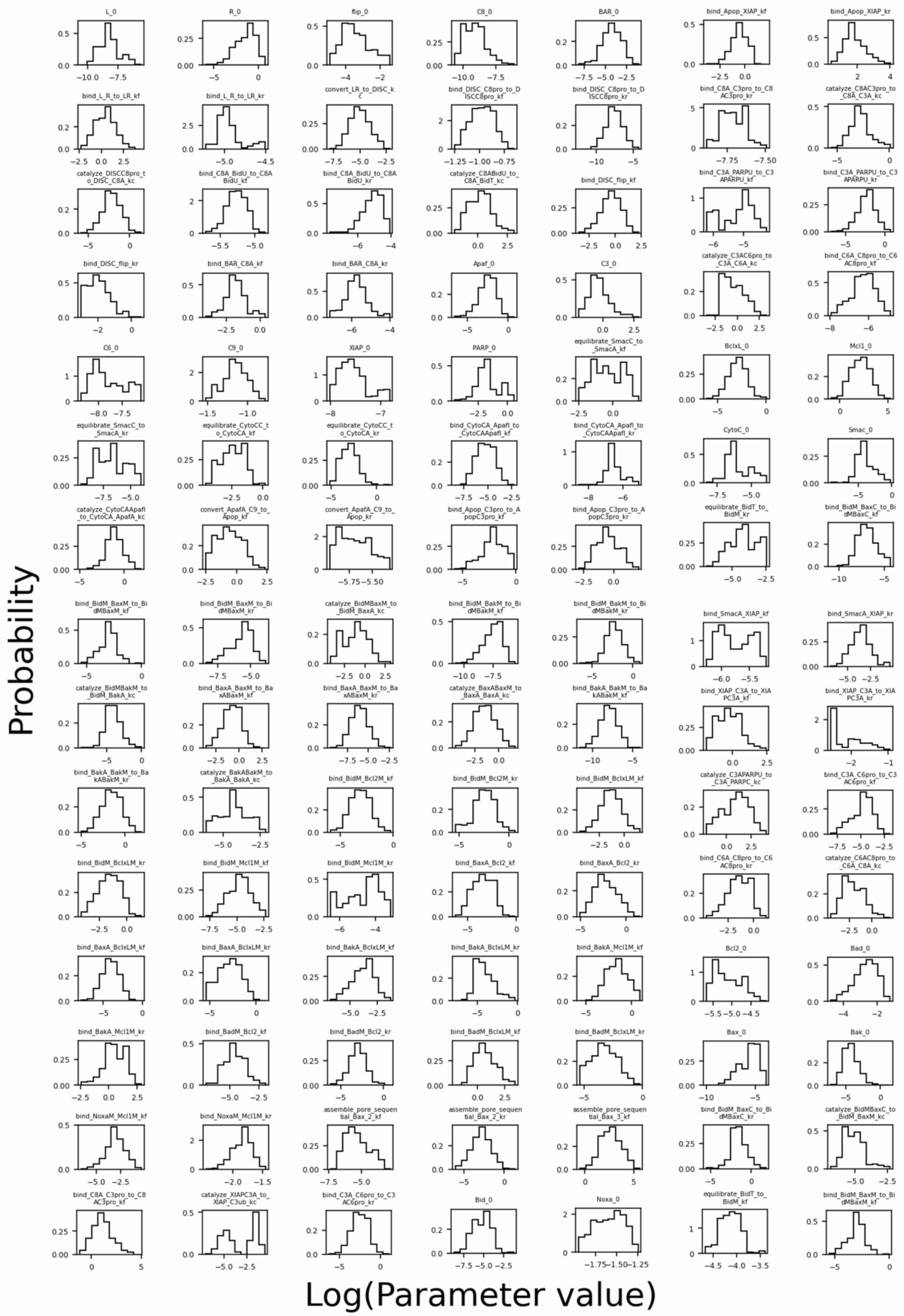

Figure S10. Marginal probability distributions of EARMv2.0 individual kinetic parameters, related to Supplemental Section 2. *Distributions were recovered from the PyDREAM run by integrating all other dimensions.*

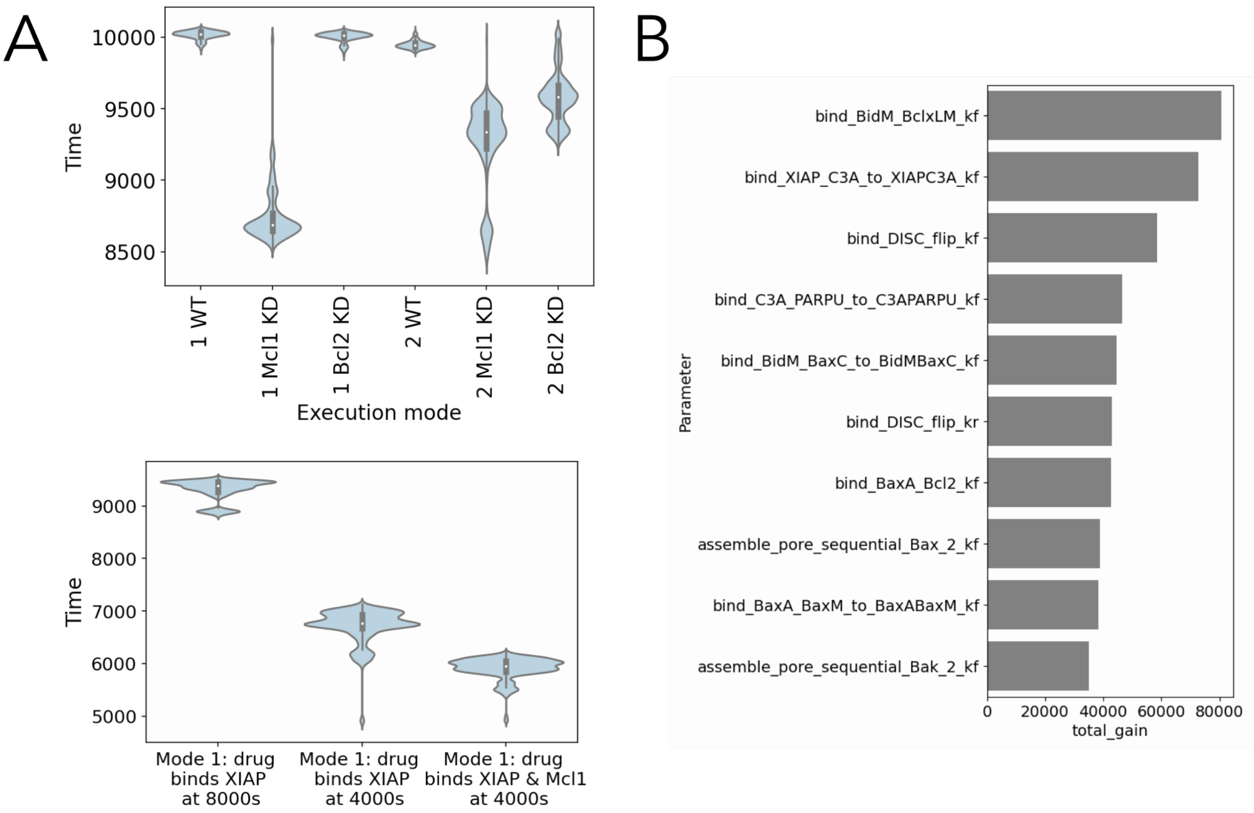

Figure S11. In silico experiments to probe the different execution modes in EARMv2.0, related to Supplemental Section 2. *A) Upper panel: Time to death distributions in the execution modes 1 and 2 in the "wild type" condition, after a 50% Mcl1 knockdown, and after a 50% knockdown of Bcl2. The boxplot inside the distributions shows the median, first quartile, and third quartile of the datasets. Execution modes 1 and 2 show substantial differences in their response to the knockdowns. Lower panel: Time to death distributions in the execution mode 1 after adding a drug that binds XIAP at t=8000 s, and at t=4000, and a drug that binds XIAP and Mcl1 at 4000 s. B) List of the 10 parameters in EARMv2.0 that contribute the most to the model prediction.*

6 Videos:

Video S1-S11: Dynamic visualization of apoptosis execution for the 11 execution modes found in EARMv2.0 obtained using PyViPR[9], related to Supplemental Section 2. Nodes represent molecular species, and pie charts inside the nodes depict relative species concentration over time. Edges represent the interaction between species, and shades of red show the intensity of the reactions.

Supplemental References

1. Shannon, C.E. (1948). A mathematical theory of communication. Bell Syst. Tech. J. 27, 379–423. http://doi.org/10.1002/j.1538-7305.1948.tb01338.x.
2. Chen, T., and Guestrin, C. (2016). XGBoost: A scalable tree boosting system. Proc. 22nd ACM SIGKDD Int. Conf. Knowl. Discov. Data Min., 785–794. https://doi.org/10.1145/2939672.2939785
3. Lopez, C.F., Muhlich, J.L., Bachman, J.A., and Sorger, P.K. (2013). Programming biological models in Python using PySB. Mol. Syst. Biol. *9*, 1–19. https://doi.org/10.1038/msb.2013.1.
4. Spencer, S.L., Gaudet, S., Albeck, J.G., Burke, J.M., and Sorger, P.K. (2009). Non-genetic origins of cell-to-cell variability in TRAIL-induced apoptosis. Nature *459*, 428–432. https://doi.org/10.1038/nature08012.
5. Shockley, E.M., Rouzer, C.A., Marnett, L.J., Deeds, E.J., and Lopez, C.F. (2019). Signal integration and information transfer in an allosterically regulated network. npj Syst. Biol. Appl. *5*. https://doi.org/10.1038/s41540-019-0100-9.
6. Gayvert, K.M, Aly, O., Platt, J., Bosenberg, M.W., Stern, D.F., and Elemento, O. (2017). A computational approach for identifying synergistic drug combinations. PLOS Comput. Biol. *13*, e1005308. https://doi.org/10.1371/journal.pcbi.1005308.
7. Sarah, C. (2017). Cancer: Identifying synergistic drug combinations. Nat. Rev. Drug Discov. *16*, 314. https://doi.org/10.1038/nrd.2017.76.
8. Kitano, H., Funahashi, A., Matsuoka, Y., and Oda, K. (2005). Using process diagrams for the graphical representation of biological networks. Nat. Biotechnol. *23*, 961–966. https://doi.org/10.1038/nbt1111.
9. Ortega, O.O., and Lopez, C.F. (2020). Interactive multiresolution visualization of cellular network processes. iScience *23*, 100748. https://doi.org/10.1016/j.isci.2019.100748.
